# Thymic Carcinomas and Second Malignancies: A Single-Center Review

**DOI:** 10.3390/cancers13102472

**Published:** 2021-05-19

**Authors:** Sunil S. Badve, Rachel Dougherty, Michael Balatico, Kenneth A. Kesler, Patrick Loehrer, Yesim Gökmen-Polar

**Affiliations:** 1 Department of Pathology and Laboratory Medicine, Indiana University School of Medicine, Indianapolis, IN 46202, USA; raeromin@iupui.edu (R.D.); michael.balatico@hsc.utah.edu (M.B.); ypolar@iu.edu (Y.G.-P.); 2 Department of Surgery and Internal Medicine, Indiana University School of Medicine, Indianapolis, IN 46202, USA; kkesler@iupui.edu; 3 Department of Internal Medicine, Indiana University School of Medicine, Indianapolis, IN 46202, USA; ploehrer@iupui.edu

**Keywords:** thymic cancer, second cancers, immune surveillance

## Abstract

**Simple Summary:**

Thymic carcinoma (TC) is a rare neoplasm that accounts for less than 0.01% of all tumors. The aim of our retrospective observational analysis is to review the incidence of second cancers associated with this histological type of cancer. We identified 92 patients with TC referred to our consultation practice and reviewed their clinical data for diagnosis of another cancer pre- or post-diagnosis of TC. This resulted in identification of 14 patients with additional cancers. The incidence of second cancer is similar to that observed in thymomas.

**Abstract:**

Thymic carcinomas account for less than 0.01% of new cancer diagnoses annually and are more aggressive than thymomas. Autoimmune disorders have been associated with thymomas and only recently with thymic carcinomas. Second malignancies are well described after thymomas. The aim of this study was to analyze the incidence of second malignancies in patients with thymic carcinomas. All cases of thymic carcinomas were identified from the pathology archives of Indiana University. Histological materials were reviewed and further correlated with clinical data to identify incidence of second cancers in patients with thymic carcinomas. Histological material was available for review in 92 cases of thymic carcinoma. Clinical data were available for 85 patients. Fourteen of these (16.5%) patients had a second malignancy; these included small cell lung carcinoma, “testicular cancer”, embryonal carcinoma, seminoma, breast carcinoma (two cases), prostatic adenocarcinoma, Hodgkin’s lymphoma, thyroid carcinoma, bladder carcinoma (two cases), renal cell carcinoma, and melanoma. The latter could precede, be concurrent with, or follow the diagnosis thymic carcinoma. The incidence of second cancers in patients with thymic carcinomas is similar to that reported for thymomas. Abnormalities in immunological surveillance may be responsible for this high incidence of second malignancies in thymic tumors.

## 1. Introduction

Thymomas and thymic carcinomas are epithelial malignancies arising from the thymic epithelium [1]. Thymomas tend to be indolent tumors with late recurrences and a well-documented relationship with myasthenia gravis [2]. The latter may be seen at presentation or at any time during the prolonged course of the disease. Impaired recognition of “self” has been postulated to lead to the development of immunologic diseases such as myasthenia. Similarly, impaired immune surveillance could increase the incidence of second malignancies in patients with thymomas [3,4,5,6,7,8,9,10,11,12,13,14]. These second tumors may precede, be concurrent with or follow the diagnosis of thymomas.

Thymic carcinoma is a very rare malignancy, accounting for less than 0.01% of new cancer diagnoses annually. The rarity of disease has been a significant limitation to understanding the natural history of the disease. Thymic carcinomas, unlike thymomas, lack organotypic features and may or may not be associated with lymphocytes. It was postulated that in thymic carcinoma, lymphocytic maturation is not impaired. A number of recent studies, including ours, have documented the presence of myasthenia in well characterized cases of thymic carcinomas [2,15,16]. A review of the ITMIG database identified that 6% of thymic carcinomas and 4% of neuroendocrine carcinomas were associated with myasthenia [2]. However, some have suggested that this could be due to a misdiagnosis of thymoma as thymic carcinoma or the lack identification of a co-existent focus of thymoma [17].

The documentation of the presence of myasthenia in thymic carcinomas suggests that immune mechanisms are abnormal in these tumors. It also raises the question of association of second malignancies with these tumors. The goal of the current study was to re-analyze a large series of thymic carcinomas for the presence of second cancers and compare the incidence with that documented in thymomas.

## 2. Materials and Methods

Ethical review and approval were waived by Indiana University institutional review Board (IRB) for the conducting of this research for this study, due to its retrospective nature. The database of patients with thymic neoplasms treated at Indiana University Simon Cancer Center was reviewed to identify patients with thymic carcinoma. The database contains patient data seen at IU over a 25-year period. The patient’s histories were searched for evidence of a diagnosis of malignancy either before, after, or concomitantly with their diagnosis of thymic carcinoma. Records were also searched for history of autoimmune disease, history of myasthenia gravis, treatment regimens and surgical management of the thymic carcinoma. The incidence of second cancers was compared to that described for thymomas as well as other cancers in the literature. Clinical data and histological findings were reviewed for all patients for whom they were available.

## 3. Results

**Figure 1:**
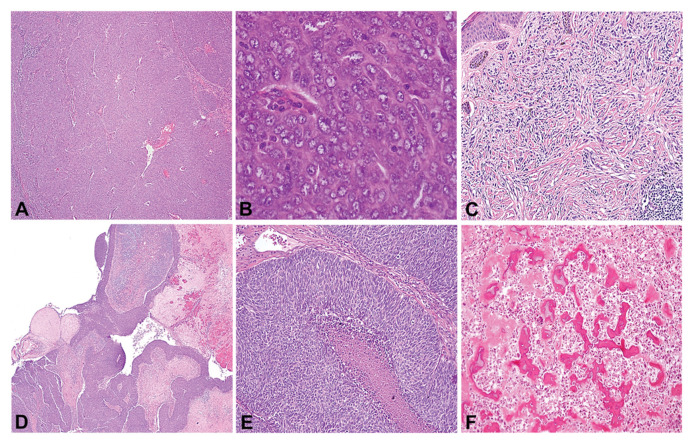
Histological features of thymic carcinomas and associated second cancers. Low (**A**) and high (**B**) magnification of a poorly differentiated thymic carcinoma that was seen in a patient with prior (2 yr) history of spindle cell melanoma (**C**). Low (**D**) and high (**E**) magnification of an unusual papillary cystic thymic carcinoma in a patient with history of osteosarcoma (**F**).

The diagnosis of thymic carcinoma is based on imaging, clinic-pathological features and exclusion of other cancers such as lung carcinoma. Histological materials from biopsy or resection specimens and detailed clinic-pathological data were available on 85 patients (Table 1). Histologically, all 85 cases were reconfirmed as thymic carcinomas. As illustrated in Figure 1A,B, thymic carcinomas are aggressive tumors associated with marked cytological atypia. Mean age was 55.4 years (range 26–79 years) with a slight male preponderance (49 male: 36 female). Two patients had Myasthenia gravis and six had other autoimmune diseases. One of these patients had autoimmune hepatitis and Hodgkin’s disease prior to the diagnosis of thymic carcinoma. Based on surgical resection and/or imaging studies, most patients were Stage III or IV as per the AJCC staging system; only one patient, each, were of stage I and II. Of the 64 patients surgically with a curative intent, only 1 had no residual. Forty-two patients had received chemotherapy only, 2 had received radiotherapy only and 37 had received both chemo and radiation. Treatment information was not available in 13 patients. Thirty patients were recorded to have metastasis during the follow-up period, ranging from 1 month to 9 years (median 2.78 years).

Sixteen second cancers had been diagnosed in 14 of these 85 patients (16.5%; Table 2). Histologically, all but one tumor were classified as poorly differentiated squamous cell carcinomas (representative in Figure 1A,B). One of the patients had an unusual papillary cystic growth pattern (Figure 1C,D). CD117 was positive in 9/10 cases and CD5 in 8/11 cases (Table 3). EGFR was negative in all cases where data were available. Eleven of these second cancers were diagnosed prior to the diagnosis of thymic carcinoma. These second cancers were of the following types: small cell lung carcinoma, testicular cancer (not specified), embryonal carcinoma, seminoma, breast carcinoma (two cases), prostatic adenocarcinoma, Hodgkin’s lymphoma, thyroid carcinoma, bladder carcinoma (two cases), renal cell carcinoma, and melanoma. One of these patients had two cancer diagnoses prior to the thymic carcinoma diagnosis. Two patients had concomitant cancers (osteosarcoma; renal cell carcinoma) and one patient had a T cell acute lymphocytic leukemia diagnosed 9 years after the diagnosis of thymic carcinoma. One patient had both a previous cancer diagnosis as well as a concomitant diagnosis at the time of thymic carcinoma diagnosis.

## 4. Discussion

The current study is the first to evaluate the rates of second malignancies in patients with thymic carcinoma. It documents the incidence of second malignancy in 16.5% of thymic carcinoma patients with available follow-up data. The association of second malignancies in patients with thymic tumors was recognized for more than 50 years [11]. In this landmark study, Souadjian et al. examined the incidence of second malignancies over a follow-up duration of 20 years following the diagnosis of thymoma. Since this initial publication, multiple studies have documented rates of second malignancies ranging from 8 to 31% particularly with thymomas of AB, B2 and B3 histology [3,4,5,7,10,14]. Pan et al. [10] observed a significantly higher risk of second cancers in thymomas (8%) as compared to that in patients undergoing thymectomy for non-tumor conditions or patients with nasopharyngeal carcinomas (2% each). Similarly, a SEER database analyses has confirmed the predisposition of thymoma patients for increased risk of second cancers (8224 per 100,000 persons) as opposed to the SEER general population (459 per 100,000 persons; *p* < 0.001) [13].

In our retrospective study, the second cancers in the majority (11/14) of patients preceded the diagnosis of thymic carcinoma; only one patient had a subsequent diagnosis of second cancer. This could represent a systematic bias as thymic carcinomas are aggressive cancers associated with poor survival.

Risk of second cancer for patients with thymoma does not seem to be correlated with age, gender, clinical history of myasthenia gravis, stage, and treatment parameters such as thymectomy and radiation therapy [4,5,7,10,14]. This may also be true for thymic carcinomas as the distribution of the preceding cancers would not have involved mediastinal radiation. However, it must be noted that treatment details for prior cancers were not available in most cases. One patient had a history of autoimmune disease prior to the diagnosis of thymic carcinoma. This confirms the results of our prior observations in the ITMIG dataset [2] with prior publications [6,16].

The incidence of second cancer after the diagnosis of the first cancer varies significantly with the type of cancer and therapeutic regimen. In patients irradiated for breast cancer enrolled in the SEER database, there was a 1% excess incidence of cancer for at least first 5 years and higher for patients receiving chemotherapy [18]. Most of the second cancers in these cohorts are endometrial cancers (due to hormonal therapy) and/or hematopoietic cancers (related to chemotherapy). The exclusion of therapy-associated cancer dramatically reduces the incidence. In a study of 40,576 patients who had survived at least 1 year after the diagnosis of testicular cancer, the incidence of second cancer was less than 5% over a period of over 50 years (1943–2001) [19]. Similarly, in an analysis of 19,068 patients with thyroid cancer in the Taiwanese cancer registry, 644 (3.4%) patients developed second cancers during 134,678 person-years of follow-up [20]. In comparison, the incidence of second cancers was significantly higher in our study.

The diagnosis of thymic carcinoma and its distinction from thymoma can be difficult. The presence of myasthenia was regarded as evidence supportive of thymoma; however, recent studies have reported this clinical feature even in thymic carcinomas [21]. The expression of CD5, KIT (CD117), EMA (MUC1), and EZH2 is frequently observed in thymic SCCs (75–85%), but is rarely expressed in thymomas (usually <5%) [22]. Of note, in the current series, one patient (#4) not only had myasthenia but also showed a lack of CD5 expression. However, histologically, this case was clearly malignant and malignancy of non-thymic origin was ruled out by clinical features as well as immunohistochemical analysis.

There are a number of limitations of the current study. Thymic carcinoma is a clinic-pathological diagnosis and is based on the exclusion of other primary and metastatic tumors, notably lung carcinoma. Indiana University being a referral center of thymic tumors, the patients being referred to it are more likely to be complicated/advanced cases. The data in this series were, in most cases, collected retrospectively. This resulted in complete clinical data being available in only 25% of patients and temporal relationships could not be documented with any degree of clarity. The lack of follow-up information in many patients could result in under-reporting of subsequent malignancies. Similarly, the possibility of biased recording of clinical history cannot be entirely excluded. Lastly, thymic carcinoma, in contrast to thymoma, is an aggressive disease, so fewer cases are likely to be identified in the post-diagnosis period.

## 5. Conclusions

This study documents an association between second cancers and thymic carcinomas. The incidence of second malignancy appears to be similar to the rates observed in thymomas. Larger multi-centric studies are necessary to confirm this association. The high incidence of second cancers makes it imperative that patients with thymic carcinomas are screened for additional malignant lesions and management decisions altered accordingly.

## Figures and Tables

**Table 1:** Clinical characteristics of the 85 patients with thymic carcinoma.

Patients with Detailed Clinical History	*n* = 85 Patients
Mean age	55.4 years (range 26–79 years)
Median age	54 years
Average greatest tumor dimension	7.26 cm (range 3.7–13 cm)
Number of males	49 (57.6%)
Number of females	36 (42.4%)
Myasthenia Gravis	2/85
Other Autoimmune disease	6/85
Received radiation only	2 (2.3%)
Received chemotherapy only	42 (89.4%)
Received radiation and chemotherapy	37 (43.5%)
Received surgery	64 (75.3%)
Patients with second malignancies	14 (16.5%)

**Table 2:** Characteristics of the 14 patients with thymic carcinoma and second cancers.

Case Number	Patient Gender	Patient Age *	Autoimmune Disease	Second Malignancy	Temporal Relationship to Thymic Carcinoma	Radiation **	Chemotherapy **	Tobacco Use
1	Male	43	No	Small cell carcinoma	Prior	No (Prior)	Yes	Unknown
2	Male	45	No	Testicular cancer, NOS	Prior	No	No	Unknown
3	Male	49	No	Embryonal carcinoma; Osteosarcoma	Prior (embryonal carcinoma), Concomitant (osteosarcoma)	No	Yes	No
4	Male	58	Myasthenia gravis	T cell ALL	Subsequent	Yes	Yes	No
5	Male	26	No	Seminoma	Prior	No	Yes	No
6	Female	64	Psoriatic arthritis	Breast carcinoma, NOS	Prior	No (Prior)	Unknown	5 pack- year history
7	Male	49	No	Follicular thyroid carcinoma	Prior	Yes	Yes	No
8	Male	75	No	Prostatic adenocarcinoma	Prior	Yes	Yes	No
9	Male	48	No	Renal cell carcinoma	Concomitant	Yes	Yes	No
10	Male	49	Yes- Autoimmune hepatitis	Hodgkin’s lymphoma	Prior	No	No	5 pack-year history
11	Female	71	No	Breast carcinoma NOS; Bladder carcinoma NOS	Prior (both)	No	No	No
12	Male	48	No	Renal cell carcinoma	Prior	Yes	Yes	Former smoker, unknown duration
13	Male	72	No	Bladder carcinoma NOS	Prior	Yes	Yes	45 pack- year history
14	Male	65	No	Melanoma	Prior	No	No	No

* Age at diagnosis of thymic carcinoma. ** If RT received for thymic carcinoma.

**Table 3:** Surgical treatment and Immunohistochemistry panel used in the case of thymic carcinoma with second malignancies.

Case.	Age	Biopsy/ Excision	CD5	CD117	EGFR	Other Pos	Other Neg
1	43	Excision	NA	NA	Neg	CK, EMA	TTF-1, AFP, HCG
2	45	Excision	NA	NA	NA	CK, EMA	TTF-1, S100, CHR, Syn
3	49	Excision	Pos	Pos	NA	CK, AE1-3	
4	58	Excision	Neg	NA	NA	CK, p63,	TTF-1, CHR, Syn, S100, MART-1, Tdt
5	26	Biopsy	Neg	Pos	NA	CK, AE1-3, p63	TTF-1, CD56, CHR, Syn, WT-1, Desmin
6	64	Excision	Pos	Pos	NA	CK,	WT-1, MOC-31
7	49	Excision	Pos	Pos	Neg	Pax-8	TTF-1
8	75	Biopsy	Pos	Pos	NA	CK, p63	TTF-1, CD56, CHR, Syn,
9	48	Excision	Pos	NA	NA	CK, p63	TTF-1
10	49	Excision	Neg	Neg	NA	CK, p63	TTF-1, PLAP, D2-40, CD31
11	71	Excision	Pos	Pos	NA	CK, p40	TTF-1
12	48	Biopsy	Pos	Pos	NA	CK, EMA, CK7,	TTF-1, Napsin, S100, CHR, Syn, CDX2
13	72	Excision	Pos	Pos	NA	CK, p63, Glut-1	TTF-1, CEA, CHR
14	65	Excision	NA	Pos	NA	CK	TTF-1, Napsin, S100, Melan-A, HMB-45

NA: Not available and/or performed.

## Data Availability

The data presented in this study are available on request from the corresponding author. The data are not publicly available due to the inclusion of patient sensitive data.

## References

[1] Abu Zaid M., Kesler K.A., Smith J., Badve S., Loehrer P.J., Raghavan D., Ahluwalia M.S., Blanke C.D., Brown J., Kim E.S., Reaman G.H. (2017). Thymoma and Thymic Carcinoma. Textbook of Uncommon Cancer.

[2] Padda S.K., Yao X., Antonicelli A., Riess J.W., Shang Y., Shrager J.B., Korst R., Detterbeck F., Huang J., Burt B.M. (2018). Paraneoplastic Syndromes and Thymic Malignancies: An Examination of the International Thymic Malignancy Interest Group Retrospective Database. J. Thorac. Oncol..

[3] Engels E.A. (2010). Epidemiology of thymoma and associated malignancies. J. Thorac. Oncol..

[4] Engels E.A., Pfeiffer R.M. (2003). Malignant thymoma in the United States: Demographic patterns in incidence and associations with subsequent malignancies. Int. J. Cancer.

[5] Evoli A., Punzi C., Marsili F., Di Schino C., Cesario A., Galetta D., Margaritora S., Granone P. (2004). Extrathymic malignancies in patients with thymoma. Ann. Oncol..

[6] Filosso P.L., Evangelista A., Ruffini E., Rendina E.A., Margaritora S., Novellis P., Rena O., Casadio C., Andreetti C., Guerrera F. (2015). Does myasthenia gravis influence overall survival and cumulative incidence of recurrence in thymoma patients? A Retrospective clinicopathological multicentre analysis on 797 patients. Lung. Cancer.

[7] Granato F., Ambrosio M.R., Spina D., Lazzi S., Rocca B.J., Voltolini L., Bongiolatti S., Luzzi L., Gotti G., Leoncini L. (2012). Patients with thymomas have an increased risk of developing additional malignancies: Lack of immunological surveillance?. Histopathology.

[8] Kamata T., Yoshida S., Wada H., Fujiwara T., Suzuki H., Nakajima T., Iwata T., Nakatani Y., Yoshino I. (2017). Extrathymic malignancies associated with thymoma: A forty-year experience at a single institution. Interact. Cardiovasc. Thorac. Surg..

[9] Owe J.F., Cvancarova M., Romi F., Gilhus N.E. (2010). Extrathymic malignancies in thymoma patients with and without myasthenia gravis. J. Neurol. Sci..

[10] Pan C.C., Chen P.C., Wang L.S., Chi K.H., Chiang H. (2001). Thymoma is associated with an increased risk of second malignancy. Cancer.

[11] Souadjian J.V., Silverstein M.N., Titus J.L. (1968). Thymoma and cancer. Cancer.

[12] Travis L.B., Boice J.D., Travis W.D. (2003). Second primary cancers after thymoma. Int. J. Cancer.

[13] Weksler B., Nason K.S., Mackey D., Gallagher A., Pennathur A. (2012). Thymomas and extrathymic cancers. Ann. Thorac. Surg..

[14] Welsh J.S., Wilkins K.B., Green R., Bulkley G., Askin F., Diener-West M., Howard S.P. (2000). Association between thymoma and second neoplasms. JAMA.

[15] Nakajima J.I., Okumura M., Yano M., Date H., Onuki T., Haniuda M., Sano Y., Yoshino I., Asamura H., Yoshida K. (2016). Myasthenia gravis with thymic epithelial tumour: A retrospective analysis of a Japanese database. Eur. J. Cardiothorac. Surg..

[16] Li W., Miao Z., Liu X., Zhang Q., Sun L., Li P., Liu W., Zhang L. (2016). Thymic carcinoma patients with myasthenia gravis exhibit better prognoses. Int. J. Clin. Oncol..

[17] Roden A.C., Yi E.S., Cassivi S.D., Jenkins S.M., Garces Y.I., Aubry M.C. (2013). Clinicopathological features of thymic carcinomas and the impact of histopathological agreement on prognostical studies. Eur. J. Cardiothorac. Surg..

[18] Matesich S.M., Shapiro C.L. (2003). Second cancers after breast cancer treatment. Semin Oncol..

[19] Travis L.B., Fosså S.D., Schonfeld S.J., McMaster M.L., Lynch C.F., Storm H.H., Hall P., Holowaty E.J., Andersen A., Pukkala E. (2005). Second cancers among 40,576 testicular cancer patients: Focus on long-term survivors. J. Natl. Cancer Inst..

[20] Lu C.-H., Lee K.-D., Chen P.-T., Chen C.-C., Kuan F.-C., Huang C.-E., Chen M.-F., Chen M.-C. (2013). Second primary malignancies following thyroid cancer: A population-based study in Taiwan. Eur. J. Endocrinol..

[21] Zhao Y., Zhao H., Hu D., Fan L., Shi J., Fang W. (2013). Surgical treatment and prognosis of thymic squamous cell carcinoma: A retrospective analysis of 105 cases. Ann. Thorac. Surg..

[22] Chan J., Chen G., Molina T.J., Ströbel P., Travis W.D., Brambilla E., Burke A.P., Marx A., Nicholson A.G. (2021). Thymic Carcinoma. WHO Classification of Tumours -Thoracic Tumours: International Agency for Research on Cancer.

